# Discussion of Papers Read at Southwestern Michigan Dental Society at Battle Creek, April 9–10, 1901

**Published:** 1901-07-15

**Authors:** 


### DISCUSSION OF PAPERS READ AT SOUTHWESTERN MICHIGAN DENTAL SOCIETY AT BATTLE CREEK, APRIL 9-10, 1901.

### DISCUSSION OF DR. BLACKMARR'S PAPER.

Dr. S. M. White, of Benton Harbor: There is one point for which dentists are to be blamed, and that is the unnecessary extraction of the deciduous teeth; but they are not entirely to blame, as much of this is done by medical doctors, but not so much now as it used to be. It used to be quite common to hear one say, "Oh, this is a baby tooth, and don't amount to anything," and they took it out, consequently the mouth was put in a condition for an irregular set of permanent teeth. The value of retaining the deciduous teeth for the full period is one of the most important matters we can teach our patients.

Dr. A. C. Runyan, of South Haven: I have been very much interested in this paper and have always taken some interest in the subject. In my humble way I have tried with the material at hand to bring about corrections of irregularities. I have used the principles advanced by Professor Angle, and made a study of them when first introduced. I believe his is the most thorough and systematic method of regulating. There are a number of good points brought out in this paper which should be emphasized. The extraction of teeth in the anterior portion of mouth in order to make space, for example the cuspid in order to make space for the lateral incisor, is wrong. This is sometimes done at the earnest demand of parents, who will, should the dentist decline to extract, call upon the family physician to do it. Another bad practice is the extraction of the temporary molars when they are decayed and the child is suffering with the toothache. By so doing the bite is allowed to come too close together and the permanent molars can not fully erupt. These are matters which should be carefully considered, and parents should teach children to go to the dentist at as early an age as two years, and thus form a habit. It will be more easy to manage them in later years, and work can be done very much better. In most instances the child is not brought to the dentist until an extraction is necessary and this frightens it and necessarily causes pain. All of these things might be prevented by a system of family education along the lines mentioned by Dr. Blackmarr in his paper.

Dr. F. H. Essig, of Dowagiac: I would like to ask Dr. Blackmarr a question. Is it practical for the regular practicing dentist to undertake all cases of regulation, and also will he explain to us how we can reach the mass of people?

We get only the well-to-do people, or those who are able to pay for an extensive operation, the poor come only for extractions. I remember a case of a young lady of seventeen years of age, who was attending the literary department at Ann Arbor; her baby teeth had been extracted at an early age, as fast as they decayed; her permanent teeth were badly irregular. It was my first case; it did not cost her anything as it was done in the college; it had not been done before because of cost. It is always too much for those who are working for common laboring wages to pay. If we had specialists in this line to whom we could send patients and be sure they would not be robbed it would be all right.

Dr. Warboys, of Albion: There is one point in reference to speaking to parents about their children's teeth. We are too liable to say, "I would not extract that tooth now but leave it alone, it will be all right, it may come out of its own accord," etc. As Dr. White has said they may go to the medical doctor, who will pull it out. When we make a statement of any dental operation it should be done in a positive manner; let parents know it is best for the child, and that we understand what we are talking about, and then they will have all the confidence which is necessary. Of course, we can speak to the little child easy-like, but by speaking to parents in a business-like manner we will surely accomplish better results.

Dr. Blackmarr closes discussion. *Mr. President:* —I am much pleased with the interest which has been taken in this paper; I feel very well paid for presenting the subject. I first became interested in this work by having patients thrown upon my hands when Dr. Case left Jackson for Chicago. I afterward took a course under Professor Angle.

In reference to apparatus. There are three classes of them, and they are subdivided into different forms. They will correct any irregularity if intelligently applied. The older the patient, however, the longer the time necessary to accomplish work.

In reference to the questions of Dr. Essig. Any dentist who will study along this line will be able to do this work. I would not say, however, that an ordinary practitioner would be able to take all the cases which may present and carry them through successfully, unless he had taken special training in such work. I do not devote my entire time to it, but a good portion of it. If Dr. Essig wishes to send me a few of his cases I will be glad to receive them.

In reference to poor children, these should be taken at an early age, as a little attention at the proper time will avoid much trouble and expense later on. Poor people all spend money, but it is just a question whether they spend it for what is going to do them the most good. When people do not appreciate this work I should certainly not undertake it. I do not think the question of whether a man is rich or poor cuts as much figure as the man himself. A poor person may be very neat in his appearance and spend money to have the teeth cared for, while a rich person may neglect his personal appearance and spend money on other worthless things.

---

Discussion on Dr. Abell's paper, see page 220, May REGISTER.

DR. S. M. WHITE, of Benton Harbor: I wish to compliment the essayist on his paper; it is short, but has, however, covered the field of dentistry from the cradle to the grave.

The ghost of my first fillings or first rubber plates will never haunt me, for the patient has either died or the other dentist has fixed him up. If I was a young man starting out into practice I would have my operating room underground or somewhere away from my reception room so when a person steps in he will not be disturbed by a complaining patient. I do not think the time will ever come when I will know what to do in every case which presents. To my mind the best thing to do is just to use the best judgment we can command, and never worry about results. I do not know of any better way to get a basis for good judgment in treatments and operative methods than to attend meetings of the dental societies.

The point of educating the public comes up at every dental meeting, and I hope it will continue to haunt all dental societies until a law is passed requiring school children to present a certificate to the teacher stating their teeth are in good condition.

The ghost of departed opportunities does not worry me, as I try to make all I can out of this life. A young man who has good business qualifications need have no fears.

The rubber ghost does not worry the dentists to-day so much as formerly as the so-called dental parlors, which make the best set of teeth for $5, have taken that class of patients off our hands.

In reference to ghost at margins of fillings I have often wished some method or treatment might be discovered which would place the cavity in a condition that would make it impossible for caries to recur. As to the root cavity ghost, various methods and treatments have been suggested from time to time, and it surely seems that from all these a perfect method of treatment and filling could be obtained which would place us in a position to cope with this ghost. This one has probably worried dentists more than any other.

Dr. F. H. Codding, of Dowagiac: There was one point in the paper which is not quite plain to me, and that is in connection with the rubber ghost. We are unfortunate enough not to have a dental parlor in our vicinity. The essayist stated that he first made a plain plate, vulcanized as thin as possible and used this as a base plate upon which he obtained the bite, and set up teeth and then revulcanizing. I do not see that anything is gained by this method over the use of the Ideal base plate, and it requires much more work. This is not a criticism, merely a question.

Dr. Blackmarr: A great many of these ghosts can be cleared up; there is an old quotation, "The cure of all fear is knowledge." This suggests a good deal. Combine good business principles with knowledge, and avoid many of these ghosts. I think dentists promise too much; we think too much of our work. You all know how straight out-and-out most business men will answer you in reference to anything in their line. If you are not satisfied and come back again the business man does not care; it is a matter of business. We should handle our patients in much the same manner; of course, we can not handle children in such a way. Do not allow patients to argue with you about work, what is best, etc.

Dr. E. A. Honey, of Kalamazoo: I am much of the same opinion as Dr. Blackmarr. I think I promised too much when I first started out in practice, and as a consequence my patients expected too much of the work. I am very careful about this now, and if a question is asked as to length of time a filling will last I simply state to them that the work is done to the best of my ability, and the length of time which it may last will depend more upon conditions over which they have control, than on the quality of my work. A patient came to my office about two years ago, a young lady of from fifteen to twenty years of age, for examination of her teeth; there were no cavities and some nice work in her mouth; she was then using milk of magnesia as a mouth wash. Did not see her for two years until quite recently and there were several cavities in her teeth and a number of the fillings were surrounded by decay and these had been put in by a careful operator. She told me she had discontinued the use of the milk of magnesia shortly after visiting me last, thinking everything was all right. Had she continued the use of the mouth wash the cavities might not have been in her teeth. Of course, a large number of fillings fail through no fault of the dentist or patient either, but in such cases we should remind them of the importance of taking the utmost care of the teeth. If I warrant a filling to last five or twenty years I can not blame the patient for coming back if the filling fails before the stated time. In mouths which have predisposing conditions for caries, it is well to caution such patients and request their aid in saving the teeth, for the fillings will not last only in proportion as the mouth toilet is given it proper care. Within the last year I have had less criticism, at the same time I know I have been making no better fillings.

DR. WARBOYS: There is one ghost which we would never see if we all had the ability and opportunity that one of whom I speak possessed. He put in a filling for a man once. After excavating the tooth he placed something in it and with hot air blew out smoke, and when he had the cavity cleaned out he pounded and pounded the gold; finally the filling was completed and the Doctor said, "When you get up there and Saint Peter asked you who put in that filling, you tell him Watling." So I say, if we all had such ability and material to work upon, we should seldom see the ghost at the margins of the fillings.

Dr. F. H. Essig: Dr. Black states that decay comes periodically, different periods of time in different mouths. If that is the case, it would be well for dentists to study each patient and try and ascertain when this period arrives so as to adopt preventive measures.

Dr. Abell, in closing the discussion. *Mr. President:*—In reference to point brought out by Dr. Codding about making small plates of rubber for taking bite, this small body of rubber does not change in shape, which is the case with wax and other things. And we find that it usually fits; it can be filed and fitted to the mouth very easily, then bite can be obtained upon it and the plate made. In case it does not fit, through some imperfection of impression or some undiscovered peculiarity of mouth it is much easier to make over than a fully vulcanized plate with teeth. The Ideal base plate is good, but does not take the place of this kind of a bite plate.

In reference to business matters of dentists, people think that dentists' work ought to be absolutely perfect; they will forgive a mistake in a business man, but not in a dentist; they expect perfect work for perfect dollars. It is hard to convince the public that it is possible to make a mistake, and still be righteous. What dentist has not been asked, "Do you warrant your work?" The people have been educated to believe we do perfect work, and if anything does occur we are to blame.

There is a feeling among young dentists who start out in practice that they know more than the old practitioners, but fortunately they soon find out there is a lot to learn; that is, most of them do. The remedy, however, for all these things rests with us, as has been brought out in the discussion.

Discussion of Dr. Gilmer's paper on "Surgical Treatment of the Antrum," see page 271, June REGISTER.

DR. J. A. WATLING: I do not know that I can say anything on this subject. I am truly greatful to Dr. Gilmer for this paper, and I feel amply repaid for coming, I wish there were more present to hear it. He has covered the subject very thoroughly and as he has had so much more experience than I it is impossible to add anything more to his presentation. I have had several of these cases and they are sometimes very annoying. I have one on hand at present, a young lady who lives at a distance from my office. I have dismissed it cured several times, only to have it break out again; she recently wrote me that it was steadily improving, but she imagines there is still a slight odor from it occasionally. In these cases which give so much trouble I have found it very advantageous to change remedies. I have in mind a case of a young married lady, whose tooth, a bicuspid, had been treated for abscess; her husband went out of town, he was a dentist. She came to my office on Saturday. When she came into the office I noticed her lip was swollen and as soon as I touched her face she jumped. I immediately decided there was disease there and advised her of the facts and told her it would be necessary to extract the tooth and I thought the trouble would be relieved, but she would not consent until her husband came home, so I gave her morphine. Sunday she was in bed, and she said she was just as happy as could be. On Monday the tooth was extracted and in three weeks there was no trouble. I had no difficulty in keeping an opening for drainage; in three weeks' time it was all healed up. I do not meet with cases very often which give in as easy as that.

Dr. Taft: I suppose one of the embarrassments in treating affections of this kind is to obtain a correct diagnosis. There is a great variety of these conditions, and in order to introduce and carry a correct treatment one must have a thorough knowledge of the condition of the disease which may exist; it may vary from mere engorgement of cavity, the natural opening being closed by inflammation of lining membrane and the secretion within the cavity being shut in, this often causes no particular discomfort except a sense of fullness, may cause bulging of eye by upward pressure upon floor of orbit, but little or no pain on external surface. There may be a very severe engorgement with much pain. All that is necessary in such cases is to open into cavity and get thorough drainage and wash out with sterilizing washes.

Again, this collection of secretion may undergo a degeneration and be a source of irritation to the tissues around about it. If the membrane becomes thickened to a great extent, which usually follows and is in a chronically discharging condition, then the curetting of cavity would be indicated. Not only will there be a thickening of the lining membrane but the cavity will be filled with a serous or muco-serous fluid and polypi will form and may occupy entire cavity, causing pressure upon cheek and pain. This is cured by opening into cavity, cutting out polypus and treating antiseptically. If the ethymoid bone is involved it is much more serious as it is so hard to reach.

Malignant growths also develop in this cavity. I remember the case of a lady who came to college with an affection of the antrum. We opened into antrum and found it completely filled with small granules like millet seed. The lady was sent to hospital; by an operation the entire cheek bone was removed and all affected tissues supposed to have been reached, but she died a few weeks after operation for the trouble came from the bones back of the antrum. This could easily have been mistaken for a benign case.

Diathesis to certain diseases may give different conditions, may have benign trouble running into malignant disease. The important matter in the diagnosis of these cases is to determine whether these conditions are malignant or non-malignant, by not doing so we may do just the wrong thing and make the case worse. A correct method of procedure can only be based upon a thorough knowledge of the conditions which belong to each.

Just how much dentists should deal with these cases is a question. I am inclined to think from the experience I have had that it is just as reliable to depend upon the advice of an experienced dentist as some surgeons, and more especially so than on the ordinary medical practitioner. On two different occasions I sent patients to eminent surgeons of the State of Ohio and they were both sent back, the trouble not being located. We are all much better informed to-day, and there are about as many dentists dealing with these affections as there are surgeons. Syphilitic conditions make diseases of antrum much more difficult to heal.

DR. GILMER in closing discussion. *Mr. President:*—There is nothing to add. Remarks of Drs. Taft and Watling were very good. Among us we have taken up sufficient time; unless there is some question which some one wishes to ask there is nothing further I care to say.

*Question:* A young man came into my office and could not remain long enough for an examination. I

took a broach and pushed up into affected tooth, when I pulled the broach there came an awful odor, like decayed egg. I told him the antrum was diseased and the tooth must come out. Would it be all right to use per oxid of hydrogen in such a case?

Dr. Gilmer: Unless there was a large opening it would be dangerous to use the per oxid of hydrogen. It was probably one of those cases where the roots nearly penetrated through the floor of the antrum, and probably what would have been necessary would have been to get rid of tooth.

